# Restoring Balance—A Woman’s Expressions of Lived Experience of Everyday Life During a Period of Aging: A Case Study

**DOI:** 10.1177/00469580231167133

**Published:** 2023-04-10

**Authors:** Margaretha Norell Pejner, Staffan Karlsson

**Affiliations:** 1 Örebro University, Örebro, Sweden; 2 Halmstad Municipality, Halmstad, Sweden; 3 Halmstad University, Halmstad, Sweden; 4 Kristianstad University, Kristianstad, Sweden

**Keywords:** experience, everyday life, aging, loss of spouse, case study

## Abstract

Bereaved older people face stressors from the changes in roles associated with the death of a spouse. To illustrate the lived experience of everyday life during a period of aging after a woman’s loss of her spouse. One woman born in 1918 was followed between 74 and 80 years of age after her husband died. Data consisted of daily diary. The text from the diaries were analyzed with a phenomenological hermeneutical approach. Everyday life after becoming a widow is characterized by balancing between personal resources to manage everyday life and vulnerability. In health and social care, it is important to identify experiences of vulnerability because these are associated with poor health.

**What do we already know about this topic?**
Bereaved older people face stressors from the changes in roles associated with the death of a spouse.
**How does your research contribute to the field?**
Everyday life after becoming a widow is characterized by balancing between personal resources to manage everyday life and vulnerability.
**What are your research’s implications toward theory, practice, or policy?**
In health and social care, it is important to identify experiences of vulnerability because these are associated with poor health.

## Introduction

Aging differs depending on gender, ethnicity, and environmental factors and can be described both as involving physiological, psychological, and social changes. The physiological changes can lead to impaired ability such as impaired vision, hearing, or mobility^1,2^ and interviews with older women in a previous study found that everyday life change in different ways. Duties women had done when they were younger could be difficult to perform, which may imply a need for help. The need of help was perceived as limiting in everyday life, but simultaneously women stated that they accepted the situation and saw it as a natural part of aging.^3^ Psychological changes due to aging can mean that the amount of life experience increases.^4^ It appears that the opportunity to make one’s own decisions and own one’s existence is important.^5,6^ Aging also means losses of relatives and friends as well as difficulties in maintaining relationships.^3^

Family members and close others can facilitate psychological recovery by moderating bereavement and enhancing empowerment and control over one’s life. This connectedness involves both interpersonal relationships and social inclusion, which ultimately contribute to restoring a positive identity.^7,8^ In addition, social networks help surviving partners organize and carry out tasks in daily life, encouraging them to adapt and move on to the new stage in their life.^9^ While social relations can also provoke negative effects, previous research has found their benefits to outweigh any negative consequences of social interaction.^10^

In the loss of a partner, bereaved individuals face stressors associated with changes in roles because of the death.^11^ Widowed persons face financial, family, administrative, and practical difficulties as well as shifts in their identity. Therefore, it is crucial to better understand the daily experiences of widowed people and to identify the coping strategies that reduce the potential negative psychosocial and physical health consequences of bereavement.^12^ The Dual-Process Model of Coping with Bereavement (DPM)^13^ integrates the Cognitive Stress Theory of Lazarus and Folkman^14^ with traditional grief theories. The DPM was specifically developed to address 2 categories of stressors and their corresponding bereavement-related coping strategies when dealing with the death of a spouse. According to the DPM, primary stressors are directly linked to the loss of the deceased person, and they include situations in which the bereaved is confronted with the loss of the relationship and the bonds to the attachment figure. Exposure to such stressors can occur as external events, such as a conversation or a memory, or as an inner experience, such as self-generated memories of the death. It also includes the painful and uncontrollable emotions (eg, longing and loneliness) that arise from that loss. Such stressors are called *loss-oriented stressors*. Secondary stressors that arise because of the loss are related to the altered life after the loss and involve stressors such as financial or household problems, new practical or social skills that need to be developed, and shifts in identity, roles, and relationships. These stressors are referred to as *restoration-oriented stressors*.^13^

Social ties and social-relational factors related to the transition to widowhood become more central to well-being both at the instrumental and emotional level after late-life stressors such as spousal loss. Social ties articulate a system of human relations that enhance psychological resilience by providing social support (both perceived and actual), applying social influence (eg, norms and social control), and offering social engagement and person-to-person contacts in addition to facilitating access to a range of resources.^15,16^ However, having social ties per se does not enhance mental health; rather, it is the social network’s characteristics (eg, composition) and, above all, the perceived quality of the social network, that determine its protective effect against depression.^17,18^ Wider and more diverse social networks, such as those not exclusively composed of relatives, but also friends, colleagues, and/or neighbors, are associated with higher levels of happiness, confidence, and self-esteem.^19^ Further, frequent contacts with close confidants support a higher level of satisfaction with one’s social network with a related positive impact on mental health.^20^

Swedish national policy encourages older people to live in ordinary housing as long as possible, which most older people also want.^21^ In Europe, about 40% of women 65 years and above are living alone.^22^ For older people, the risk of experiencing loneliness could be related to the fact that people who are important to them, for example, partners, older relatives, or friends, are dying to a greater extent in old age. The opportunity to find a new partner or new friends also decreases with increasing age. When one’s social network decreases, that is, social contacts and exchanges with other people outside the home, the risk of loneliness increases.^23,24^

Late life spousal bereavement is mainly faced by women.^25^ Widowed women are found to be 75 years of age in average, and they typically live another 15 years after the loss of their spouse.^26^ Research indicates that bereavement is harmful to health and well-being.^27,28^ In particular, the first 2 years of bereavement are crucial and are closely related with negative impact on health.^29,30^ Spousal loss may influence the older widows’ physical and mental health and may worsen existing age-related health problems.^31,32^ The perspective of bereavement has mostly focused on decline in health and wellbeing, risk factors, and illness. Generally, it was assumed that to get over the loss of the spouse it was first necessary to do “grief work,”^33^ emphasizing the importance to all bereaved individuals of working through negative thoughts, memories, and emotions after a loss. The transition process was a linear process moving in stages toward the resolution of the painful experience.^34^ However, research has shown a lack of empirical support for this. Previous research indicates that the transition to widowhood, despite the risk of developing negative effects on health and well-being, is experienced by many widows with a high degree of resilience,^35-37^ and at least 40% of the bereaved adapt well with no noticeable changes in well-being during the bereavement transition.^35^ However, there is room for a better understanding of managing daily life from the perspective of the older widows.

## Purpose

The purpose of this study was to illustrate the lived experience of everyday life during a period of aging after a women’s loss of her spouse.

## Research Design

### Design

To be able to capture the unique situation with its complexity and to understand it over time we used a case study. The case study gives the opportunity to see the story of a specific individual and many smaller cases in the single case.^38^

### Sample and Data Collection

Case studies allow for different kinds of data, for one or more cases, and for inductive or deductive approaches. In this case study the data consisted of a woman’s diary. The woman was born in 1918 in the north of Sweden and began to write a diary when her husband died in 1990. The diary entries from the 2 first years had been lost, and thus the data consisted of diary entries written between 1992 and 1998, which meant she was between 74 and 80 years old. The woman kept a daily diary and everyday notes that consisted of 75 to 100 words describing everyday life, the persons she met, and the activities she performed. The text from the diaries was extracted verbatim prior to the analysis. One of her sons handed over the diaries to the authors with the hope that an analysis of them could increase the understanding of the lived experience of everyday life during a period of aging after the loss of a spouse.

### Analysis

**Figure 1.**
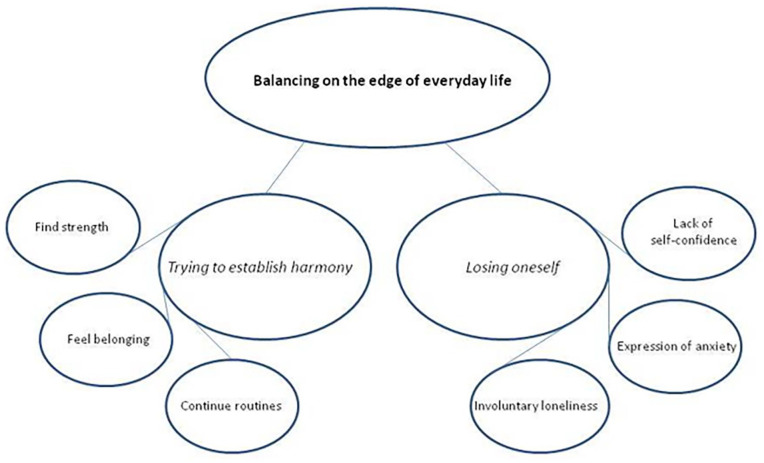
Theme with respective categories and subcategories.

Within a phenomenological hermeneutical method for interpreting texts, a naive understanding of the text is formulated from an initial reading. The text is then divided into meaning units that are condensed and abstracted to form sub-themes, themes, and possibly main themes, which are compared with the naive understanding for validation. Finally, the text is again read as a whole, the naive understanding and the themes are reflected on in relation to the literature about the meaning of lived experience and a comprehensive understanding is formulated.^39^ In this study, the data were analyzed using Lindseth and Norberg’s^39^ version of phenomenological hermeneutical analysis inspired by Ricoeur. Philosophy according to Ricoeur includes critical hermeneutics in empirical studies that are focused on communication where bridges are built between what may appear to be contradictory positions. Ricoeur believes that concrete time indications and existential experiences of time can be brought together into what is called the historical time, the time in which we live and work. An interpretation according to Ricoeur is presented as a field in which the tension between explaining and understanding is at the center.^40^ The analysis follows 3 stages in which one moves in a dialectical motion between understanding and explanation. First, in the *naïve reading*, the authors read the text individually several times to understand its meaning as a whole. The authors also discussed the content of the text several times, including its structures and formulations, in order to find a consensus. Second, in the *structural analysis* one of the authors put together different pieces of text, so-called meaning units that described the same events, and interpreted them into sub-themes (Figure 1). Both authors together discussed how the meaning units related to the sub-themes and to the research question and how they could be reflected into themes and a main theme. Third, to achieve a *comprehensive understanding* the sub-themes, themes, and main theme were reflected against the research question and the data. The text was read again by both authors, and its trustworthiness was discussed.^41^ At this stage the results were also evaluated at a seminar where other researchers and research students participated.

## Ethics

The data does not contain personal data that needs ethical review according to Swedish Law on Ethical Review.^42^ Information cannot be traced to individuals and names have been changed to fictional names in the results. Geographic locations have been excluded. The data (diaries) has been personally delivered from the widow’s family to both authors after having email contact with them. The first author has personally returned the original diaries to the family. A copy of the material is kept safe at NN University as research material.

## Results

The diaries testified to how the experiences of being a widow oscillated between the 2 main categories of *“Establishing* harmony” and *“Losing oneself,”* which resulted in the theme *“Trying to restore balance in everyday life.”* The main category *“Establishing harmony”* was described by the subcategories *“Continuing routines,” “Feeling belonging,”* and *“Finding strength,”* while the main category *“Losing oneself”* was described by the subcategories *“Involuntary loneliness,” “Expressions of anxiety,”* and *“Lack of self-confidence”* (Figure 1).

### Trying to Restore Balance in Everyday Life

This theme declares that everyday life is like a balancing act between coping with and not being able to manage everyday life.

*“Don’t* *know how I should be so that it will be good (June 5 -93)”*

*“Thank you and forgive me for complaining (March 25 -96)”*

Trying to restore balance appears to be on the edge between succeeding in finding harmony and getting lost in the new life.

### Establishing Harmony

This main category was about managing everyday life based on the new conditions regarding what it means to be a widow. The fundamental goal was to preserve everyday rhythms by accomplishing the well-known, the things that she had always done felt feel safe and comfortable with, as illustrated by the subcategory *“Continuing routines.”* Trying to establish order was also about managing the unknown, the new conditions that it meant to be a widow, and how to make it a part of everyday life as illustrated by the subcategories “*Feeling belonging”* and *“Finding strength.”*

#### Continuing routines

The diaries told us that everyday chores not only maintained meaningfulness in everyday life, but also described life’s rhythm over time. Everyday order consisted of maintaining the ordinary, the things with which one feels confident with and always have done. These things

Further, the diaries described the people she met, where she went if she was in need of new clothing, and how she got to the store.

Over time the diaries show the rhythm of her life because events were recurring, not only what happened during the week or month, but also over holidays, seasons, and years. Like the weather, the events were described with a comment that valued the effort made or the feeling of how she had managed it. The holidays were characterized by the same routines and procedures such that one can follow them almost to the day from year from year.

*“Took out the Christmas tree today, been out shovelling snow, hard work (January 14 -92)”*

*“Took out the Christmas tree today, been out shovelling snow (14 Jan-93)”*

*“Tried to take out the Christmas tree and it went well (January 15-95)”*

*“Shall now take out the Christmas tree and Christmas things (January 15-96)”*

In the autumn, the leaves should be raked and the boat taken out of the lake. When the snow falls, it must be shoveled, and the grass must be cut in the summer. The efforts were mostly commented on with an evaluation of whether it went well, was hard, or how it affected her mood.

*“I also scrubbed the porch railing and then painted it. It was hard, but happy that I managed (July 1 -94)”*

*“Then I have been out cutting bushes and grass. Hard work (June 6 -93)”*

*“Mowed the whole lawn, it went well (July 3 -96)”*

#### Feel belonging

The diaries described the social context the widow was in, the different persons around her, their names, and where they lived. She had daily contact with several of them, and they visited each other over lunch or a cup of coffee. They also helped each other with shopping, going to the doctor, or with practical things at home. They had a lot in common with each other, and the diaries revealed how the company was valued and expressed gratitude of being with them.

*“It has been so much fun to have been here and to have company all day (January 6 -93)”*

*“It was a fun trip. Glad I got to go with Gustav, Sara and Viktor. Safely. We got home at 5 o’clock. I’m* *a little tired now but* *it’s been so much fun. Thank you for my nice boys tonight (March 26 -95) ”*

*“Kalle came and wanted me to hem the pants he bought (March 29 -95)”*

As a part of belonging, the diaries revealed that there also was a confirmed loneliness when it was not possible to be together with others. This could be evaluated as feelings of loneliness or of being lonely during a period of time. One can find a distinct difference between when one is together with somebody and when one is not together with them. When one is not together with them, there is a feeling that they are still there. Disappointment was expressed if visits by friends did not take place.

*“Has been a lonely day, no one has been here (1 Jan, 3 Jan, etc. -93)”*

*“Alone most all day (February 6 -94)”*

*“Sigurds did not come today. They were out in Borge′s boat on the lake (July 29 -95)”*

#### Finding strength

Beyond maintaining routines and having a sense of belonging, the diaries testified that establishing order in everyday life was a consolation. This was done by prayer to God. The prayer was sometimes quotes from a poem or text with a religious background but could also consist of short sentences related to everyday life. The prayer both gave thanks for the help received and appealed for more help.

*“You can put your burden on Jesus. You can talk to him as a friend. You may pray, and prayers will be heard. You can hope and rejoice again (April 30 -92)”*

*“Thank you for today, good Jesus (6 Dec -97)”*

*“Thank you for the help today. Good God (16 Dec -95) ”*

*“Thank you for today. Jesus, help me with the device [hearing aid], it just whines. (9 Sep-95)”*

*“Thank you for today, Good God. Help me get to sleep (15 Jan -96) ”*

The prayer was also about gratitude that the day had gone well for her own sake, but it also showed a concern for others.

*“Thank you for today, help everyone who is having a hard time Jesus (23 Oct -95)”*

Additional aspects of the prayer regarded concerns about not being enough, as if one had not done one′s best to establish order. The conversation in the prayer included thankfulness on the one hand and apologizing for negative behavior on the other.

*“Thank you for today Jesus. Forgive me if I did something wrong (18 / 1-96)”*

*“Thank you and forgive me for complaining (March 25 -96)”*

Finding strength included the feeling of being needed, being useful, or being there for someone else and by doing something for another person.

### Losing Oneself

This main category was about realizing one’s vulnerability and fear of not being able to cope with everyday life and how it affect it. The subcategories gives expression to the lived experience of an undesirable situation and the feeling of inability to get out of it. The loneliness has different aspects and changes over time that appears in the subcategory different *“Involuntary loneliness”*. Even anxiety in the subcategory *“Expressions of anxiety”* expressed in different ways, both related to physical symptoms as to the inexplicable and indescribable. *“Lack of self-confidence”* is about having to step into new roles and the uncertainty this brings.

#### Involuntary loneliness

The diaries told us that there was a lived loneliness, which was so difficult to deal with that it took all of her energy. The loneliness was something she did not want, and it was hard to know how to get out of it. There was also an expression of the need for help in escaping the loneliness and to be happy again.

*“Loneliness is difficult (28 Feb -92)”*

*“The loneliness is worse (21 Jan -93)”*

*“Help me so that I can cope with the loneliness (24 Sep -92)”*

*“How should I be happy again, loneliness (11 Aug -92)”*

Initially the diaries described that the loneliness was about regret for her husband. It was a loss of someone who had been close to her and now no longer existed. Somebody that she needed and that she could not understand how she could live without. The loss became painful, difficult to deal with, and meaningless. The painfulness expressed itself in bodily symptoms and also led to a sense of lack of initiative.

*“Difficulty with loneliness and anxiety in the chest. Miss my Erik every day (1 Jan -92) ”*

*“I have difficulty with loneliness, I miss my Erik so much, more and more (6 Aug -92)”*

*“I miss my Erik so much, loneliness is so difficult, meaningless, forcing me to everything (9 Sep -92)”*

Besides the lived experience of losing her spouse in a permanent way through death, it appeared in the diaries that there was also a present fear of being left behind, or being de-prioritized by someone she wanted to continue a friendship with. The reason for being de-prioritized perhaps lied on unresolved conflicts with a friend. The friendship could be preserved, but the risk of failing led to experiences of psychological stress.

*94)”*

*“Thinking of Tyra, do not want to become unfriended by her (7 Sep -93)”*

*“Feeling bad from unfriendliness, hope it works out (24 Sep -93)”*

*“How will it be good with Tyra again (20 Sep -93)”*

*“It’s unpleasant that Tyra says that about me. Hope it will be good. Getting*

*nervous (22 Jan-94) ”*

#### Expressions of anxiety

From the diaries, it appeared there was an underlying concern that was constantly present both when alone and when in the company of others. It also revealed that there were some broodings, but it was not clear over what. There were expressions regarding nervousness and anxiety in general, but also in relation to health problems and to other persons.

*“Slept better, but worried (July 9 -93)”*

*“Feeling so nervous, just thinking. Agnes also came in. Hope all the worries are resolved (19 Aug -93) ”*

*“Been down to Ingrid. She did not feel well. Neither do I (20 Feb -94) ”*

*“I’m scared. Help (July 27 -95) ”*

The anxiety also manifested itself in physical symptoms such as palpitations, headaches, or dizziness.

Despite medical examinations that stated that there was nothing serious, there was still a feeling that something was not right—a feeling that something changed in the body but was not recognized. There was an appeal for help and at the same time an uncertainty about being able to obtain it. Situations occurred in which measures could relieve physical as well as mental health problems, which could alleviate the anxiety.

*“Worried about the heart, became dizzy. Unpleasant. Help me (April 5 -93)”*

*“Feeling anxious, my head hurts so much and it irritates my ears, it’s hard. How should I get help (18 Jan -93)”*

*“I am worried. Buzzing and ringing in the ears (14 Jan -96) ”*

*“Was dizzy and worried in the chest, have been lying down for quite a long time, feeling better now (13 Aug -97)”*

#### Lack of self- confidence

The diaries told about feelings of concern or a feeling of not being able to cope with things or with practical issues—things that she had not done before because another person had done them, and now she must deal with them herself or needs to get help from someone to handle the situation. This raised a fear of having to expose herself to something that she could not manage.

*“Called Ludvig who told me how I should screw on a nut, and it was good. I get so scared when something breaks (14/2-94)”*

Further concerns were about taking responsibility for things she had not taken responsibility for before. Sometimes it was like a new world had opened up to her and she did not know if she could dare to take responsibility for it. Experiences of fear and anxiety were expressed, and confirmations from others could bring a feeling of safety in the situation.

*“Afraid and worried, help. Have written a bank transfer myself. Therefore, I wanted Ingrid to check that I did the right thing. It was good (23/1-96) ”*

## Discussion

Mourning processes can end up in an undesirable situation that poses challenges in everyday life. The results presented here showed that everyday life after the loss of the person’s spouse involved maintaining a balance by using personal resources (*“Establishing harmony”*) and meeting one’s vulnerability (*“Losing oneself”*). Personal resources (*“Establishing harmony”*) described in the result can be compared with what Antonovsy^43^ describes in its salutogenic model “generalized resistance resources” (GRRs). Antonovsy^43^ means that the person is located somewhere in a movement on a continuum between health, well-being/ disease, dissatisfaction. GRRs is the property of a person to cope with stressors that a situation with disease, vulnerability involve. The purpose is to maintain balance in existence.

Bailey^44^ indicates that the ability to maintain or recapture balance in life is a personal and innate resource that comes naturally to human beings. The return to balance occurs through the emotional state of consciousness referred to as serenity, in which a person can look at their situation objectively and thus can gain the ability to act in order to change their situation. It appears that the ability to get into a serene state can decrease in connection with disease or illness, which can make it difficult to regain balance in everyday life (Bailey^44^), and persons with long-term disease often experience that they end up in an imbalanced state in everyday life caused by their disease and they use different strategies to regain control and to restore balance in everyday life.^45-48^ The diaries in this study did not describe the presence of long-term disease that could be the cause of balancing between one’s resources and vulnerability, but instead described other life events that led to difficulties in maintaining balance in everyday life. Obbia et al^49^ mention frailty as a form of vulnerability. Frailty includes both pathological and psychosocial constituents where a triggering event can affect the balance of everyday life. Such a trigger can be changes in family life, like becoming a widow. For health and social care staff, it is important to identify symptoms at an early stage because frailty is a predictor for poor health.^50^ Preventive interventions for older people should not only focus on morbidity, but should also take into account other influencing life events.

Mourning work consists of 2 sides. The diaries initially told us that there was a pronounced longing and grief after her husband, which could be interpreted as a part of the loss-oriented stressors process. The diaries also described positive events in everyday life that—based on the DPM—might be parts of the restoration-oriented stressors process. This may imply that mourning work was going on and that there were different strategies for dealing with it. Stroebe and Schut^13^ argue in their DPM that in connection with a loss everyday experiences oscillate between loss-oriented and restoration-oriented processes as part of the mourning work. The mourning work is seen as a normal reaction and is expected to last for weeks to months before adapting to the new life situation.^51^ Stroebe and Schut^13^ point out that strategies in relation to the restoration-oriented process often consist of changes or introductions of new events in everyday life. Maintaining routines only makes sense if they are important.^52,53^ Davies et al^53^ concluded that some persons stop performing routines in connection with becoming a widower because they are connected to the person they have lost and the meaning of the routine thus disappears. The persons continue performing other routines that are considered meaningful. Whether the routines described in the diaries were new or not is not apparent, but it might be reasonable to believe that year-round and daily routines described in the diaries were important and could be part of strategies described in the restoration-oriented process. In the diaries it seemed that the acute grief work with a pronounced yearning after her husband weakened over time, which is a natural development in the grieving process.^51^ Further, the diaries told us that there was almost daily social participation with neighbors, friends, and next of kin, which indicated that there was an active social network. It appears that to be actively seeking social contexts is a coping strategy to recovery from an acute episode of illness and to restore balance in everyday life.^49^ In addition to restoring balance, participating in social activities can also be a strategy to cope with loneliness.^47,53^ More research is needed to further explore loneliness and social participation and its association with frailty in older people suffering from grief.

Psychological complaints and challenges in everyday life can trigger feelings of discouragement. The results showed that involuntary loneliness was expressed and described by the woman in her diaries. Involuntary loneliness could be related to Yanguas et al^54^ description of emotional loneliness. Loneliness in old age is often associated with frailty, but the concept is multidimensional and complex. Yanguas et al^54^ sum up the literature and distinguish between emotional and social loneliness. The emotional loneliness is described as the absence of someone to share thoughts and feelings with, while social loneliness is the lack of social contacts and a social context with which one can feel a sense of belonging. Stanley et al^55^ mention another form of loneliness, namely self-chosen loneliness. Such loneliness offers peacefulness and time for reflection and harmony, unlike the emotional and social loneliness that can lead to frailty. However, social loneliness was expressed in the category *“Feeling belonging”* as being part of a social context even though not being there presently. This result was not consistent with other studies’ descriptions of loneliness^54,55^ because the results in this study indicated neither a lack of social contacts nor that the loneliness was self-chosen. Loneliness was found in this study, but whether it was accepted or not was unclear. To explore various types of loneliness, research is required to develop methods for assessment in clinical practice.

### Limitations

Using a phenomenological approach with a hermeneutical analysis was appropriate because the method allowed participation in the diaries narrative.^39^ In the participation, one can take part in the woman′s history and convey an experience of it. However, some methodological aspects must be mentioned. The credibility of the study could be questioned because the diaries were not data collected in a scientific manner, and when they were written there was no intention to use them in a future scientific context. The texts were concise and written with a dialectal language that may have provided opportunities for misinterpretations. There has neither been the possibility to ask additional questions nor to receive confirmation that the text has been understood correctly. On the other hand, data consisted of natural expressions that had been written down spontaneously without anyone asking questions. This was a strength, as the data collection was without influence from research questions or an interviewing researcher. The data consisted of spontaneous images from everyday life. The sample size, one case study, can also be a limitation as transferability cannot be ensured. However, the detailed description of the method and the authors’ experiences of qualitative research methods may have strengthened the credibility of the study. The stability of the data collection with diaries over a long time could strengthen the dependability, but a weakness is that the authors had no influence over the data collection. Discussions between the authors during the analysis process to achieve consensus supported the confirmability of the study.

The results might have a certain measure of transferability to older widows after the loss of a spouse. The study used longitudinal data, but with the weakness that the data covered only one person living in a specific context.

## Conclusion

Everyday life after becoming a widow is characterized by balancing between the personal resources needed to manage everyday life and the vulnerability of everyday life. The resources include the ability to continue to carry out meaningful routines, have contacts with one’s social network, and find comfort in one’s faith. The vulnerability is the realization of not being able to cope with situations in everyday life along with feelings of anxiety and loneliness. The feeling of loneliness was twofold. The involuntary loneliness was a sign of vulnerability, while the perceived loneliness was a sign of belonging and was seen as a resource. In health and social care, it is important to identify at an early stage when feelings of vulnerability take over because these are associated with poor health. This also requires in-depth knowledge of the different emotions that are associated with vulnerability and how to counteract these emotions.
